# *Sedum alfredii SaNramp6* Metal Transporter Contributes to Cadmium Accumulation in Transgenic *Arabidopsis thaliana*

**DOI:** 10.1038/s41598-017-13463-4

**Published:** 2017-10-17

**Authors:** Shuangshuang Chen, Xiaojiao Han, Jie Fang, Zhuchou Lu, Wenmin Qiu, Mingying Liu, Jian Sang, Jing Jiang, Renying Zhuo

**Affiliations:** 1 State Key Laboratory of Forest Genetics and Breeding, Xiangshan Road, Beijing, 100091 P.R. China; 2 Key Laboratory of Tree Breeding of Zhejiang Province, The Research Institute of Subtropical of Forestry, Chinese Academy of Forestry, Hangzhou, 311400 China; 3 Chemical Biology Center, Lishui Institute of Agricultural Sciences, Lishui, Zhejiang Province 323000 China; 4 Biotechnology Research Center of China, Three Gorges University, Yichang, Hubei 443002 China

## Abstract

The plant natural resistance-associated macrophage protein (Nramp) family plays an important role in tolerance to heavy metal stress. However, few *Nramps* have been functionally characterized in the heavy metal-accumulating plant *Sedum alfredii*. Here, *Nramp*6 was cloned and identified from *S. alfredii* and its function analyzed in transgenic *Arabidopsis thaliana*. *SaNramp6* cDNA contains an open reading frame of 1, 638 bp encoding 545 amino acids. *SaNramp6*′s expression can be induced by cadmium (Cd) stress, and, after treatment, it peaked at one week and 12 h in the roots and leaves, respectively. *SaNramp6* localized to the plasma membrane in protoplasts isolated from *A. thaliana*, *Nicotiana benthamiana* lower leaf and onion (*Allium cepa*) epidermal cells. The heterologous expression of *SaNramp6* in the *Δycf1* yeast mutant increased the Cd content in yeast cells. *SaNramp6* also rescued the low Cd accumulation of the *A. thaliana nramp1* mutant. Transgenic *A. thaliana* expressing *SaNramp6* exhibited high Cd accumulation levels, as determined by a statistical analysis of the Cd concentration, translocation factors and net Cd^2+^ fluxes under Cd stress. Thus, *SaNramp6* may play a significant role in improving Cd accumulation, and the gene may be useful for the biotechnological development of transgenic plants for phytoremediation.

## Introduction

A well-balanced cellular concentration of essential metals such as iron (Fe), copper (Cu) and manganese (Mn), plays a fundamental role in the normal growth and development of plants^1^. However, the absorption of heavy metals such as lead (Pb), cadmium (Cd) and arsenic (As), can upset the normal metabolism within plant cells and also cause damage to human and animal health through the cumulative effects of the food chain. For example, Cd is a carcinogenic factor closely related to the generation of breast and kidney cancer^2,3^, and high levels of Pb toxicity can lead to irreversible damage to the central nervous system^4^. The ever-increasing worldwide contamination of soil and water by heavy metals is a problem that demands a prompt solution^5^. Phytoremediation is presently regarded as an eco-friendly and cost-effective strategy to clean heavy metal-polluted soils with the help of hyperaccumulating plants^6^. Among the more than 400 naturally hyperaccumulating plants, an ecotype of *Sedum alfredii* that co-hyperaccumulates Cd, Zn and Pb was first found in China^7,8^. Previous physiological studies suggested that this ecotype is a promising hyper-accumulator for the decontamination of polluted soils, because it can accumulate up to nine g of Cd per kg of leaf dry weight (DW)^9–11^. However, the detailed molecular mechanism underlying its hyperaccumulation and tolerance of heavy metals is still unclear. Taking advantage of this genetic resource for the breeding of future phytoremediation-associated plants requires a functional analysis of potential heavy metal-responsive genes in the hyperaccumulating ecotype of *S. alfredii*.

Metal transporters are essential for the maintenance of appropriate metal ions concentrations within different cellular compartments^12,13^. Among the identified metal transporters, natural resistance-associated macrophage protein genes (*Nramps*) are considered to play potentially important roles mediating metal ion homeostasis at multiple cellular levels in plants. First cloned in mouse, *Nramp* gene family members are relatively evolutionarily conserved throughout organisms, including plants, animals, yeast and bacteria^14^. The *Nramp* genes comprise a small family represented by six members in *Arabidopsis thaliana* ^15^, 12 members in rice (*Oryza sativa*; http://www.ncbi.nlm.nih.gov/gene/? term = Nramp + Oryza + sativa), eight members in soybean (*Glycine max*; http://www.phytozome.net/soybean) and six members in poplar (*Populus trichocarpa*)^16^. Several NRAMP members have been experimentally characterized in *A. thaliana* and are involved in the uptake, intracellular transport, translocation and detoxification of metals^14,17,18^. They are all membrane spanning proteins, with the 10–12 hydrophobic transmembrane domains characteristic of metal transporters^19^. When overexpressed in yeast, *AtNramp1*, *AtNramp3* and *AtNramp4* show high affinities for Fe, Mn and Cd, whereas *AtNramp6* can transport Cd, but not Fe or Mn^20–23^. In rice, *OsNramp1* shows transport activity for Cd and Fe, but not Mn. *OsNramp4* is the first transporter identified for the trivalent aluminium ion, and the knockout of *OsNramp5* results in a significantly reduced Cd uptake^18,24,25^. *Nramp* genes have also been cloned and characterized from other plants, such as tomato (*Solanum lycopersicum*)^26^, soybean^27^ and some metal-hyperaccumulating species. A better understanding of the mechanisms used by metal transporters will provide insights into the detoxification and accumulation of toxic heavy metals in plants.

Although *Nramp* genes have been cloned and analyzed in other plant species, few studies have been reported regarding *Nramps* in the hyperaccumulating ecotype of *S. alfredii*. The transcriptome of *S. alfredii* under Cd stress indicated that an *Nramp* gene was greatly up–regulated after CdCl_2_ treatment^28^. The gene has an 80% homology with *AtNramp6*. Here, we described the isolation and characterization of the *Nramp* gene *SaNramp6* from *S. alfredii*. A subcellular localization analysis indicated that SaNramp6 is a plasma membrane transporter. Moreover, the overexpression of *SaNramp6* in *A. thaliana* increased the uptake and accumulation of Cd. Thus, *SaNramp6* may be a potentially important heavy metal-responsive gene that could be useful for phytoremediation. This work will aid in understanding heavy metal hyperaccumulation and tolerance in *S. alfredii*.

## Results

### Isolation and sequence analysis of *SaNramp6*

**Figure 1:**
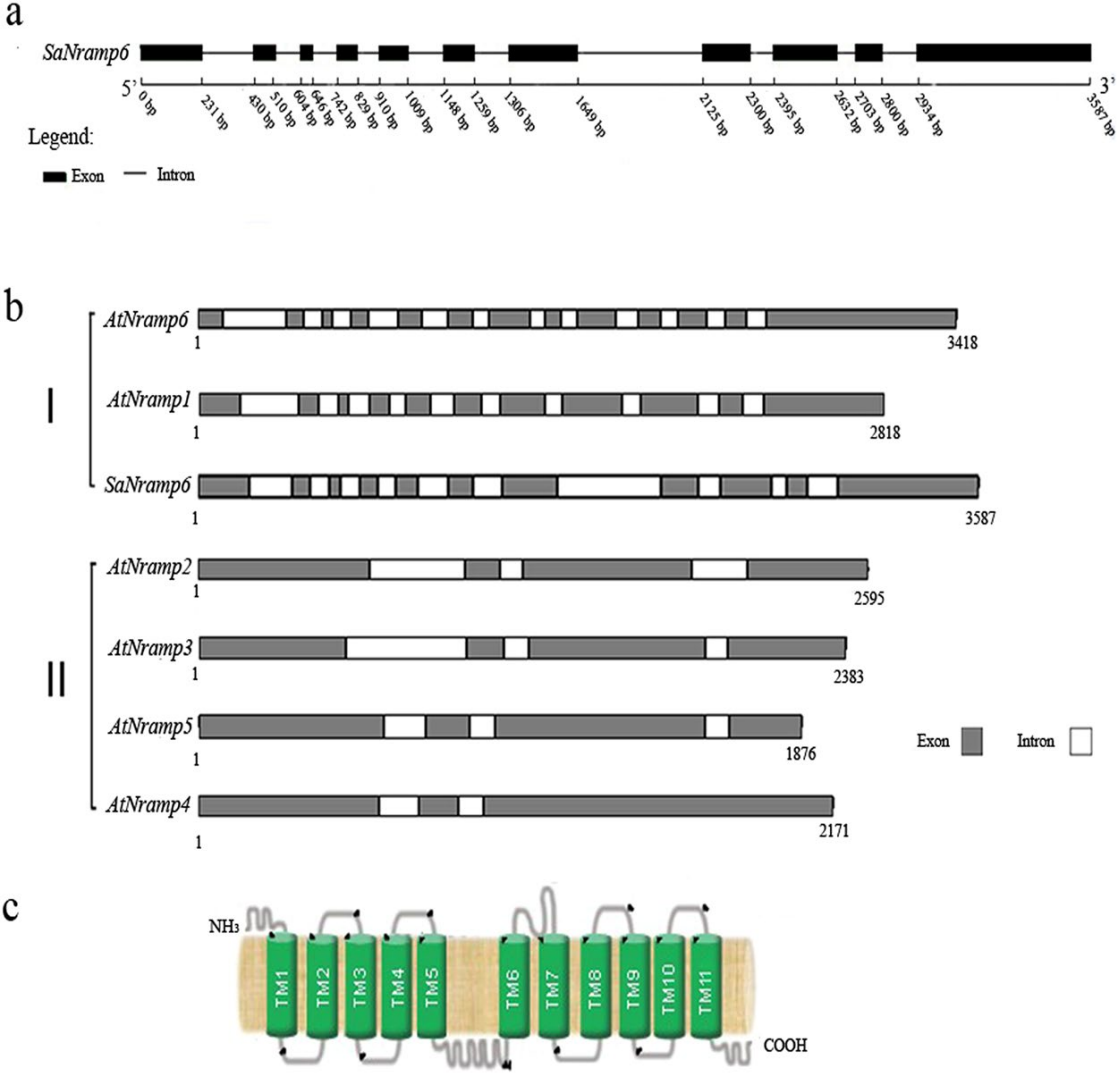
*SaNramp6* gene structure. (**a**) Genomic organization of *SaNramp6*. Black boxes and lines denote exons and introns, respectively. The numbers refer to the position between the exons and introns, (**b**) Comparison of the genomic DNA structure of *SaNramp6* and several *Nramp* genes of *Arabidopsis* available in GenBank. The white boxes represent the introns, and the grey boxes represent the exons. The numbers indicate the length of the sequence. І and II indicate the groups of *Nramps* in *A. thaliana*, (**c**) Transmembrane domains predicted by the SOSUI program. (*AtNramp1*: AT1G80830; *AtNramp2*: AT1G47240; *AtNramp3*: AT2G23150; *AtNramp4*: AT5G67330; *AtNramp5*: AT4G18790; *AtNramp6*: AT1G15960).

To identify the function of *SaNramp6* from *S. alfredii*, a full-length cDNA sequence of 2, 055 nucleotides was isolated, comprising a 1, 638-bp open reading frame, and 95-bp 5′and 322-bp 3′-untranslated regions. The specific primers *SaNramp6*-F and *SaNramp6*-R were used to amplify the sequence of the *SaNramp6* from genomic DNA to investigate the genomic structure of *SaNramp6*. The genomic sequence spanned 3, 587 bp including 10 introns and 11 exons (Fig. 1a). A sequence comparison revealed that *SaNramp6* is similar to members of group I from *A. thaliana* (Fig. 1b).

The predicted protein encoded by *SaNramp6* contained 545 amino acid residues with a putative molecular weight of 58.44 kD and an isoelectric point of 7.97. The deduced amino acid sequence was not predicted to have a signal peptide by SignalP software (ExPASy). Based on analyses using the CELLO and SOSUI programs, we hypothesized that this protein is located at the plasma membrane and has 11 transmembrane domains (Fig. 1c).

**Figure 2:**
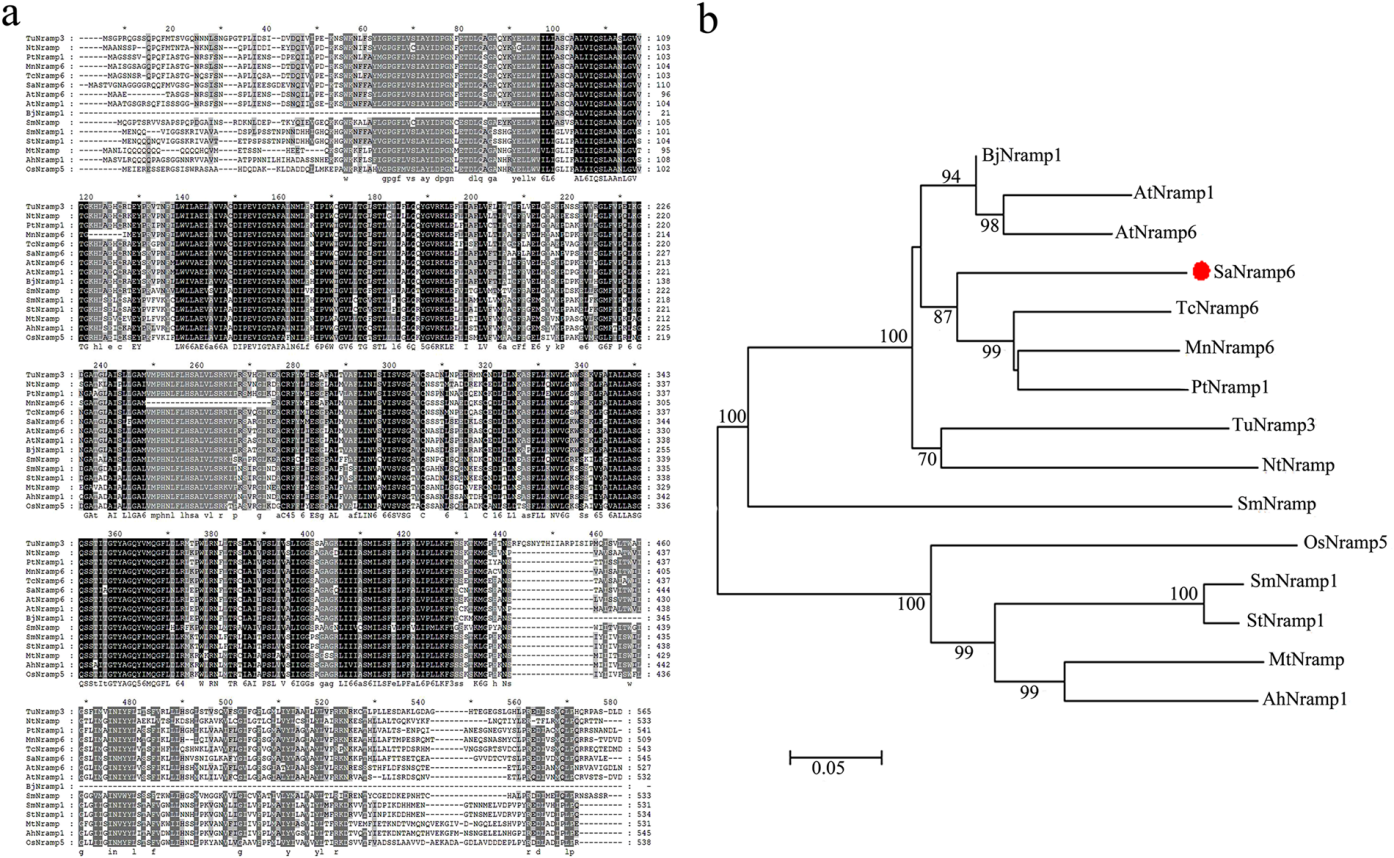
Comparison of *SaNramp6* to *Nramps* of other species based on Nramp amino acid sequences from different species. Accession numbers for sequences used are listed in Table 2.

Multiple sequence alignments with SaNramp6 revealed high levels of similarity to the Nramps of other species (Fig. 2a). To investigate the evolutionary relationships among Nramps from different species, a phylogenetic analysis was performed based on the amino acid sequences. As shown in Fig. 2b, SaNramp6 shows 80% sequence similarity to Nramp6 from *A. thaliana*, 79% to Nramp6 from *Theobroma cacao*, 78% to Nramp1 from *Populus trichocarpa*, and 71% to Nramp1 from *Nicotiana tabacum*. A phylogenetic analysis revealed that the SaNramp6 was most closely related to AtNramp6 (Fig. 2b). Based on this, we designated this gene as *SaNramp6* (GenBank accession no. KF887490).

### Expression profiles of *SaNramp6* under CdCl_2_ stress

**Figure 3:**
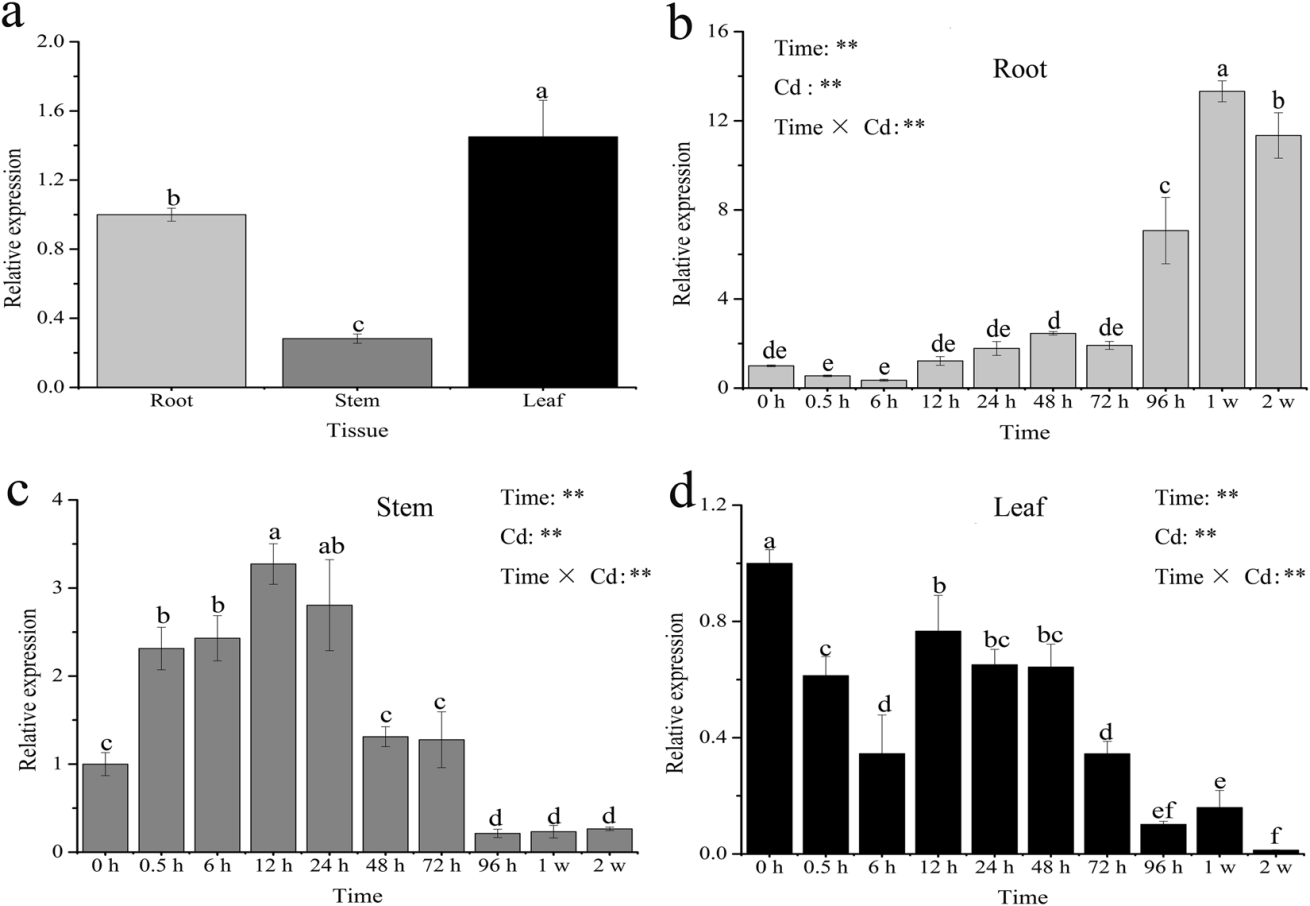
Expression patterns of *SaNramp6* in *S. alfredii* under Cd stress. (**a**) Different tissue without any heavy metal treatments, (**b**) Root, (**c**) Stem, (**d**) Leaf. The normalized mRNA levels without treatment (y-axis “Relative mRNA expression”) were set arbitrarily to 1. Bars indicate means ± standard deviations (SDs) of at least three independent biological experiments. Different letters on the bars indicate significant difference between the treatments. P-values of the two-way ANOVAs of Time, Cd (Cd treatment) and their interaction (Time × Cd) are indicated. *P < 0.05; **P < 0.01.

To examine transcriptional changes under CdCl_2_ stress, the expression of *SaNramp6* was monitored at different Cd-stress treatment times in leaves, stems, and roots. Without heavy metal treatment, *SaNramp6* was highly expressed in roots and leaves (Fig. 3a). However, the relative expression levels of *SaNramp6* varied greatly in different tissues under Cd treatment period progressed. Despite starting at a higher level, *SaNramp6*′s expression was not induced in leaves and in fact was reduced during treatment, reaching only a maximum of less than one-fold of the initial level at 12 h (Fig. 3d). In stems, *SaNramp6*′s expression increased gradually before 12 h, and then declined (Fig. 3c). By contrast, *SaNramp6*′s transcript accumulation was highly induced in roots (Fig. 3b). It began to increase within 12 h of treatment and peaked at around one week (14-fold).

### *SaNramp6*′s expression enhances Cd^2+^ sensitivity and increases Cd^2+^ content in yeast

**Figure 4:**
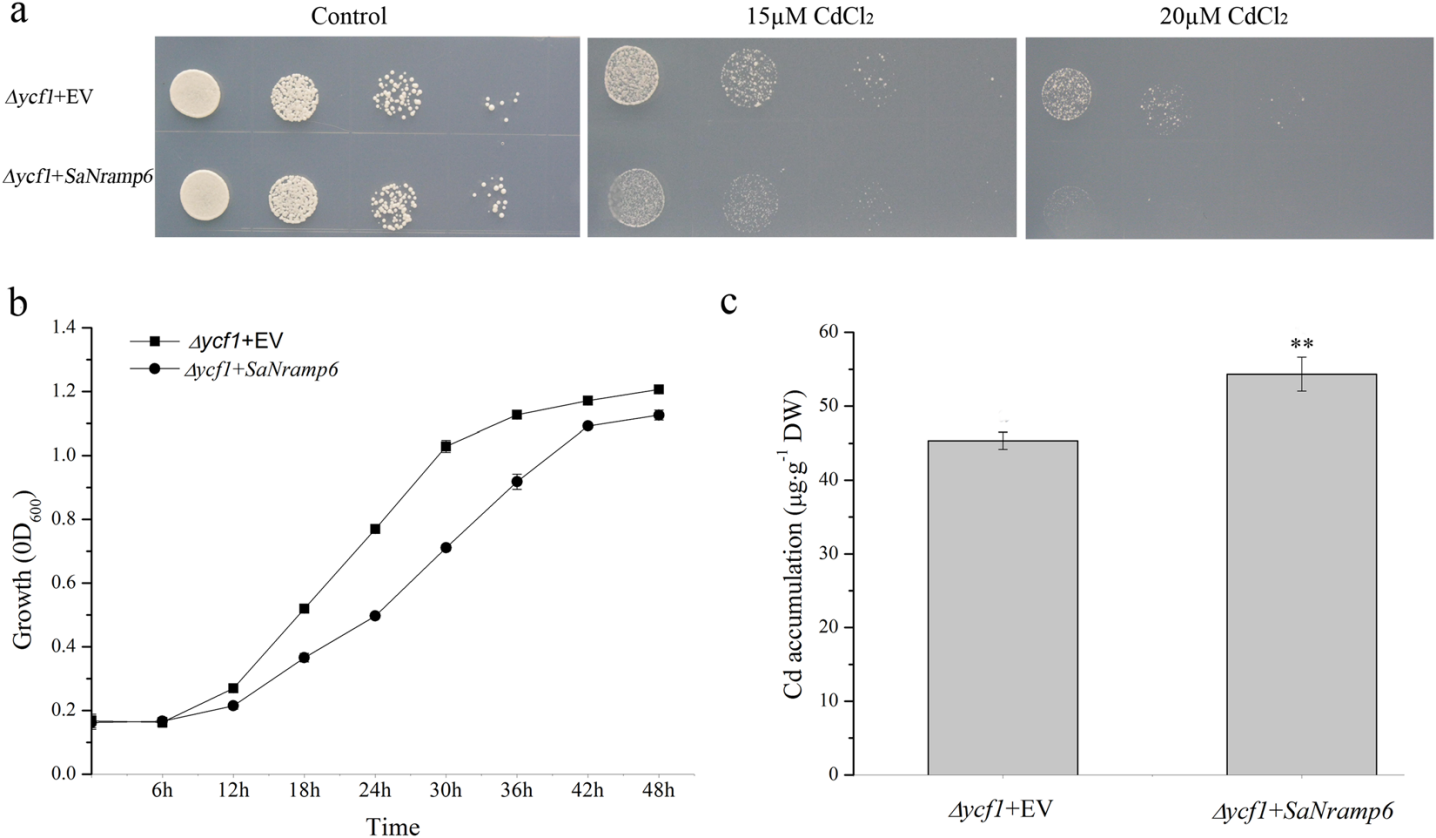
*SaNramp6* expression increases Cd^2+^ sensitivity and Cd^2+^ content in yeast. (**a**) Growth of *∆ycf1* yeast cells expressing *SaNramp6* on plates containing SG-U without CdCl_2_. (Left) or supplemented with 15 μM CdCl_2._ (Middle) and 20 μM CdCl_2_. (Right), (**b**) Time-dependent growth of yeast strains in SG-U liquid medium supplemented with 5 μM CdCl_2_, (**c**) Cd content of *∆ycf1* yeast cells expressing *SaNramp6* grown for 48 h in liquid SG-U supplemented with 5 μM CdCl_2_. Bars indicate means ± standard deviations (SDs) of at least three independent biological experiments. One or two asterisks indicate a significant difference at P < 0.05 or P < 0.01 from the *∆ycf1* + EV.

To investigate the cellular function of *SaNramp6*, the protein was expressed in *Saccharomyces cerevisiae* yeast mutant (∆*ycf1*) susceptible to Cd excess. *SaNramp6* and empty vector-complemented ∆*ycf1* cells were grown in SG-U medium overnight. Cells grown overnight were used for spotting on SG-U agar plates supplemented with 0, 15 and 20 µM CdCl_2_ at indicated dilutions. The Cd supplementation of the medium caused more considerable growth inhibition in yeast cells expressing *SaNramp6* than in the control (Fig. 4a). We also analyzed the relative growth in liquid media in the presence of Cd in yeast cells. The growth of *∆ycf1* cells expressing *SaNramp6* were lower than cells transformed with the empty vector (Fig. 4b). The growth inhibition due to the functional *SaNramp6* in *∆ycf1* suggested that *SaNramp6* may facilitate the import of Cd inside the yeast.

To test our hypothesis that *SaNramp6* may mediate the Cd uptake, the metal content was measured in yeast cells expressing *SaNramp6* or the vector that were grown in the presence of Cd. A significantly enhanced accumulation of Cd was observed in yeast cells expressing *SaNramp6* compared with the control (Fig. 4c)

### Subcellular localization of SaNramp6

Bioinformatics analysis using the CELLO v2.5 program software predicted that SaNramp6 is localized in plasma membrane.

**Figure 5:**
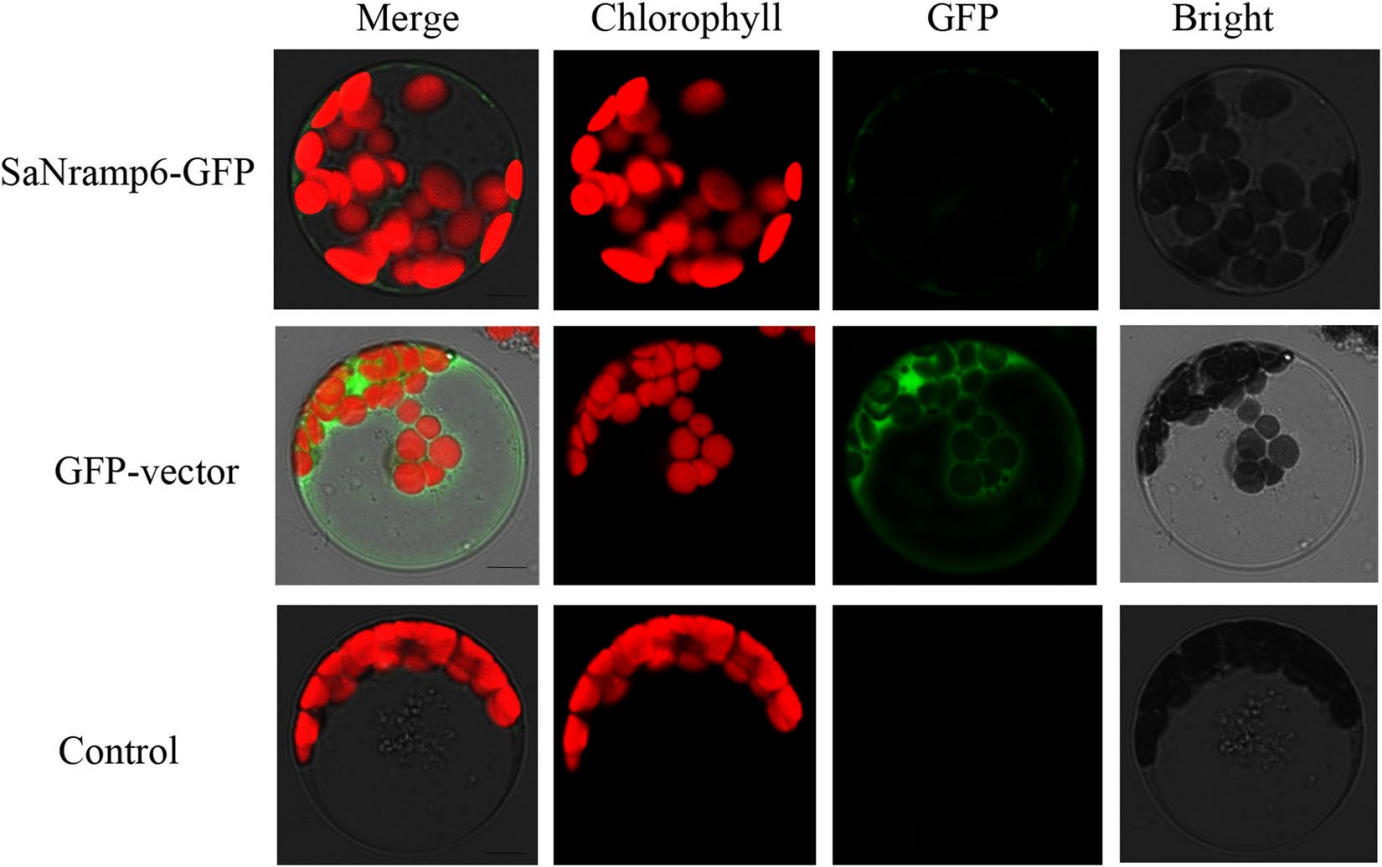
Subcellular localization of SaNramp6. Control, Non-transformed protoplast; GFP-vector, protoplast transformed with p35S-GFP vector; SaNramp6-GFP, protoplast transformed with SaNramp6-GFP fusion. Scale bar = 7.5 μm.

To test the prediction, the subcellular localization of SaNramp6 was analyzed by transiently expressing the SaNramp6-GFP fusion protein in protoplasts isolated from *A. thaliana*, onion epidermal cells and *N. benthamiana* epidermal cells, respectively. As shown in Fig. 5, visualized fluorescence indicated that the SaNramp6-GFP signal was localized at the plasma membrane, whereas the green fluorescent signal in the GFP control vector was distributed throughout the cytosol taken chlorophyll as control in protoplasts of *A. thaliana*.

The plasma membrane localization of *SaNramp6* was further confirmed by the transient expression of SaNramp6-GFP in onion epidermal cells *and N. benthamiana* epidermal cells. The fusion protein was found to be targeted to the plasma membrane by colocalization with FM4–64 within 5 min of the onset of staining (Supplementary Figure S1). These results indicate that SaNramp6 is localized at the plasma membrane, consistent with the prediction by the CELLO software.

### *SaNramp6* participates in oxidative damage in transgenic *Arabidopsis*

**Figure 6:**
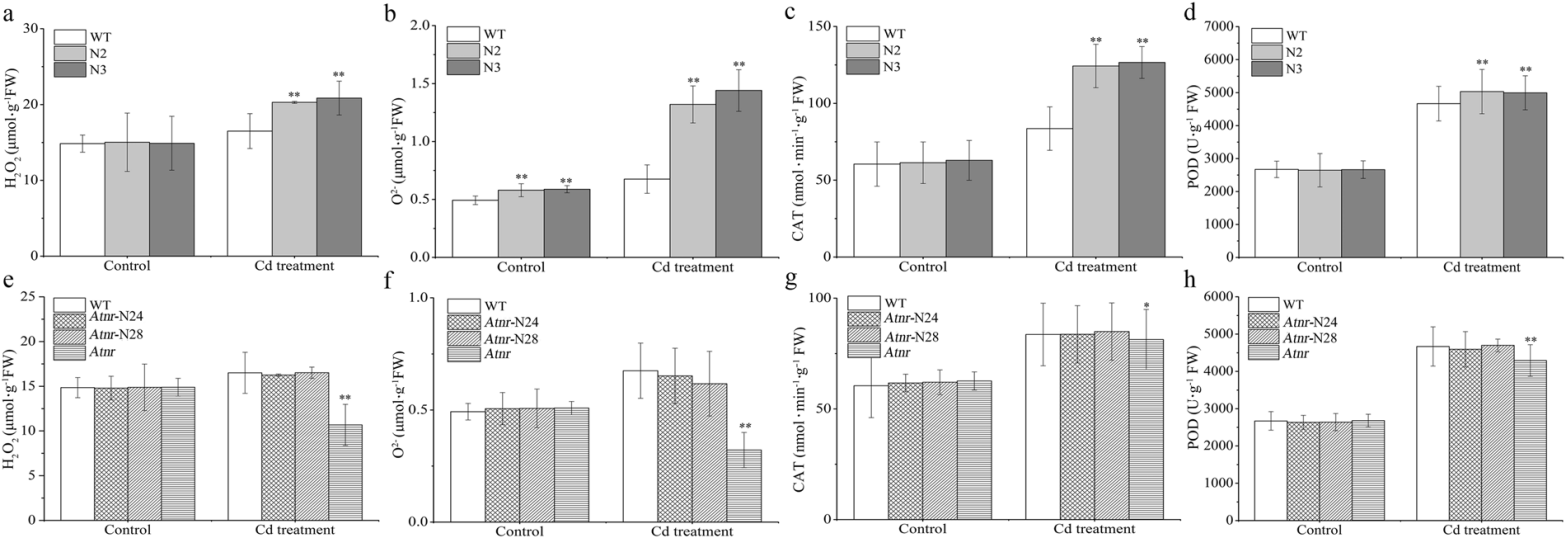
ROS accumulation responses to Cd stress and physiological indicators in four different lines - WT (wild type); OE 2 and OE 3 (overexpression lines); *Atnr* (mutant line); *Atnr*-N24 and *Atnr*-N28 (rescue lines). (**a**,**e**) H_2_O_2_, (**b**,**f**) O_2_^−^, (**c**,**g**) CAT activity, (**d**,**h**) POD activity. Control, without Cd treatment; Cd treatment, 30 µM Cd treatment for two weeks. Bars indicate means ± standard deviations (SDs) of at least three independent biological experiments. One or two asterisks indicate a significant difference at P < 0.05 or P < 0.01 from wild type.

The production of reactive oxygen species (ROS) in the different lines was analyzed using H_2_O_2_ and O_2_^−^ accumulation. As shown in Fig. 6, the contents of H_2_O_2_ and O_2_^−^ in the transgenic lines (OE 2 and OE 3) were markedly increased and both were nearly 30% higher than those in WT line. However, they were decreased or slightly increased in the mutant *nramp1* (*Atnr*) and rescue of *nramp1* lines (*Atnr*-N24 and *Atnr*-N28), respectively (Fig. 6a,b,e,f). Thus, upon Cd stress, the overexpression of *SaNramp6* could result in a high level of H_2_O_2_ and O_2_^−^ accumulation.

We next examined the scavenging ability of ROS by determining CAT and POD activities. The concentration of CAT and POD in the different lines had no difference in the control. Nevertheless, the CAT activity was dramatically increased in transgenic lines as was the POD activity under Cd treatment (Fig. 6c,d,g,h). Thus, the overexpression of *SaNramp6* caused more damage and enhanced CAT and POD activities under Cd treatment.

In addition, the four lines (WT, overexpression lines, mutant line, rescue lines) had no obvious differences in roots without Cd treatment (Supplementary Figure S2). However, the root length of transgenic lines (OE 2 and OE 3) was longer than that in the other lines in two weeks after the Cd treatment (Supplementary Figure S2).

### Overexpression of *SaNramp6* resulted in an increased Cd concentration

**Figure 7:**
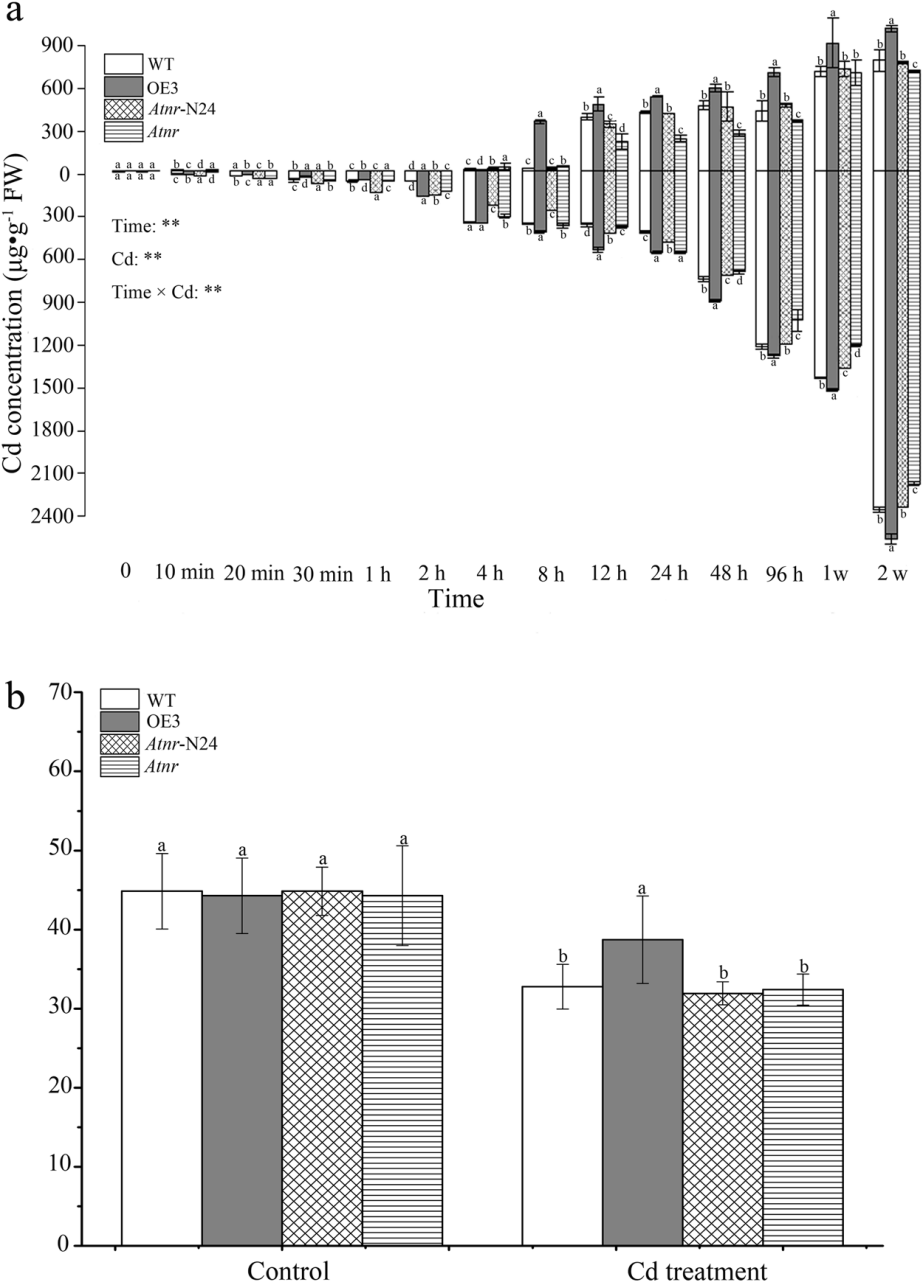
Time-dependent Cd-uptake experiments (**a**) and Cd-translocation factors (Tf) (**b**) in four lines. WT (wild type); OE 3 (overexpression lines); *Atnr* (mutant line); *Atnr*-N24 (rescue lines). Bars indicate means ± standard deviations (SDs) of at least three independent biological experiments. Different letters on the bars indicate significant difference at same time within treatments (WT, overexpression lines, mutant line, rescue lines). P-values of the two-way ANOVAs of Time, Cd (Cd treatment) and their interaction (Time × Cd) are indicated. *P < 0.05; **P < 0.01.

Time-dependent Cd-uptake experiments using aerial parts and roots were conducted to evaluate the differences in Cd-uptake abilities by the different organs of the four lines. The time-dependent experiment on the four lines (WT, overexpression lines, mutant line, rescue lines) showed that Cd concentration increased as the treatment period progressed and the pattern of Cd uptake by roots displayed an initial slower stage during the first eight hours, followed by a second, rapid stage over the subsequent two weeks and it was significant lower in OE 3 than in WT, *Atnr* and *Atnr*-N24 (Fig. 7a). The Cd concentrations in the roots of all of the lines increased remarkably under Cd-stress conditions for two weeks. However, compared with the other lines, the OE 3 had significant higher concentration of Cd in its aerial parts. Cd was transferred to aboveground parts began in 8 h and increased steadily in the following two weeks. The translocation factor of the transgenic lines was markedly higher than that of the other lines after Cd exposure for two weeks (Fig. 7b). These results indicated that the transgenic lines may have a better absorption capacity for Cd. Thus, *SaNramp6* may influence the accumulation ability of Cd in *S. alfredii*.

**Figure 8:**
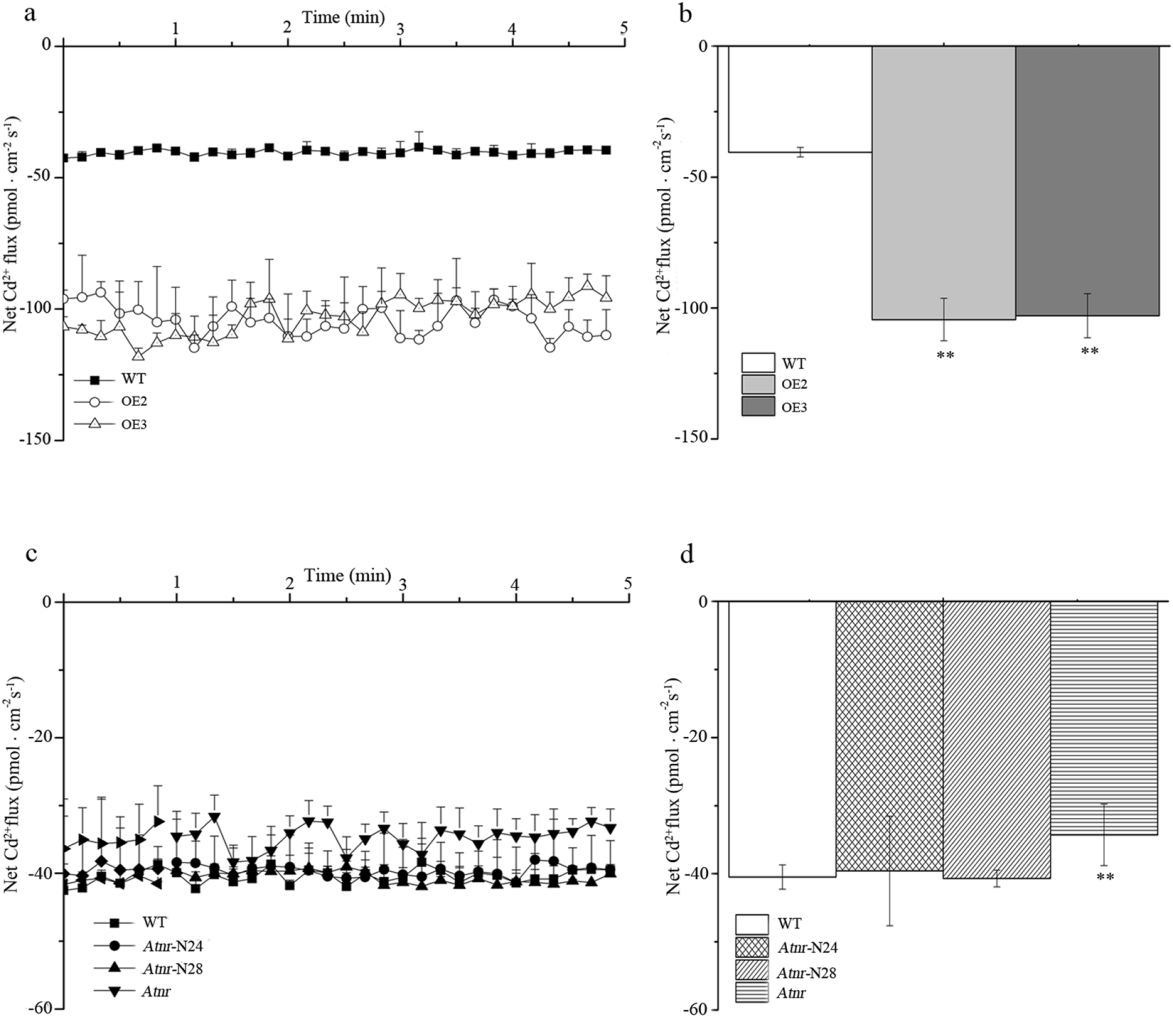
Comparison of Cd concentrations and net Cd^2+^-influx rates in four lines - WT (wild type); OE 2 and OE 3 (overexpression lines); *Atnr* (mutant line); *Atnr*-N24 and *Atnr*-N28 (rescue lines). (**a**,**c**) Cd^2+^ flux rates with Cd treatment for 24 h. (**b**,**d**) Mean flow rates of Cd2^+^. Bars indicate means ± standard deviations (SDs) of at least three independent biological experiments. One or two asterisks indicate a significant difference at P < 0.05 or P < 0.01 from wild type.

To decipher the phenomenon of Cd accumulation in the four different lines, Cd^2+^ was measured in 30 d after 30 μM Cd treatment. Significantly higher Cd^2+^-influx rates were identified in overexpression lines (OE 2 and OE 3) compared with WT line (Fig. 8a,b) and markedly lower in *Atnr* line (Fig. 8c,d); however, there were no differences between rescue lines (*Atnr*-N24 and *Atnr*-N28) and WT line (Fig. 8c,d).

## Discussion

Here, we cloned an *Nramp* family member from a heavy metal-accumulating ecotype of *S. alfredii* and the results showed that it conferred the ability to accumulate Cd in overexpression transgenic *A. thaliana*. Cd is a strongly toxic heavy metal transported across plant membranes by physiological metal transporters^29^. To date, various gene families related to the transport of Cd have been reported, such as the P-type ATPase superfamily^30^, ABC transporters^31^ and the CE family^32^. Among these metal transporters, the Nramp family is widely distributed in mammals, fungi and bacteria.

The determination of SaNramp6′s subcellular localization is important for understanding its potential roles in the process of accumulating metals. AtNramp6 is located in a vesicular-shaped endomembrane compartment and works as an intracellular Cd transporter^23^. Similarly, OsNramp1 was localized to the plasma membrane in onion epidermal cells, and the overexpression of *OsNramp1* results in a Cd accumulation in the leaves^33^. The soybean Nramp homologue, GmDmt, is located on the peribacteroid membrane of root nodules and mediates ferrous iron uptake in yeast^27^. In *Thlaspi japonicum* H., a nickel (Ni) hyperaccumulator, TjNramp4 could specifically transport Ni and increase Ni concentrations^34^. The deduced amino acid sequence of SaNramp6 shares an 80% identity with AtNramp6, and the phylogenetic tree also indicated that SaNramp6 was most similar to AtNramp6. In addition, our subcellular localization analysis showed that SaNramp6 was located in the plasma membrane. Thus, SaNramp6 could function as a metal transporter in the plasma membrane (Fig. 5).

An plasma membrane localization is consistent with *SaNramp6* conferring Cd uptake by increasing the Cd content in plant tissues. *AtNramp3* is involved in increased metal tolerance or accumulation. However, *AtNramp6* leads to Cd hypersensitivity when overexpressed in *Arabidopsis*, and *Arabidopsis* plants lacking *AtNramp6* are more resistant to Cd than WT lines^23^. In rice, *OsNramp1* participates in cellular Cd uptake or transport and the overexpression of *OsNramp1* enhances tolerance to Cd and increases Cd accumulation in shoots^24^. *OsNramp5* is a major transporter for Cd uptake, influencing Cd absorption in both solution and soil cultures^25,33^. Hence, compared with the control, *SaNramp6*′s overexpression in *A. thaliana* could accumulate more ROS of roots when exposed to CdCl_2_. We believe that understanding its functions in plants will facilitate the development of Cd-accumulating plants.

The growth of the Cd sensitive yeast strain (*∆ycf1*) transformed with the empty vector was inhibited by Cd, and yeast cells harbouring the *SaNramp6*-expression vector exhibited weaker growth activities. The results indicated that *SaNramp6* cannot complement the Cd sensitivity or rescue the Cd-sensitivity phenotype in the mutant yeast strain. However, the Cd concentration of the *SaNramp6*-expression strain was 10% higher than that of the empty vector strain (Fig. 4). The induction of *SaNramp6* expression by CdCl_2_ suggested that the gene might be involved in responding to heavy metal stress and is a transporter for Cd uptake in *S. alfredii*. Consistent results have been reported using a similar approach. Thomine *et al*.^22^ found that the growth of transgenic yeast expressing *AtNramp1*, *AtNramp3*, *AtNramp4* was strongly reduced in liquid cultures supplemented with 3 µM CdCl_2_ compared with the control, and these genes increased the Cd content in yeast. However, *AtNramp3*-OE in *Arabidopsis* were found to be hypersensitive to Cd. *TcNRAMP3*′s-expression increased Cd sensitivity and the Cd content in yeast, and *TcNRAMP3*-OE in tobacco resulted in a slight sensitivity of root growth to Cd^35^. The growth of yeast strain *Δycf1* was affected by *OsNRAMP5*, which is involved in Cd transport^25^. Therefore, these data showed that some NRAMP members could increase Cd sensitivity and Cd concentration. Here, our results from *SaNramp6* in transgenic yeast with Cd-sensitivity phenotype and Cd concentration (Fig. 4) and that in transgenic *A. thaliana* were consistent with the previous results (Fig. 7).

The capacity to reduce Cd-associated oxidation may be an important mechanism contributing to Cd uptake and transport. To test the role of *SaNramp6* in heavy metal-stress tolerance, a functional analysis was carried out by overexpressing *SaNramp6* in *A. thaliana* and rescuing the *Arabidopsis* mutant *nramp1*. In the physiological assays (Fig. 6), the root lengths of overexpression transgenic plants were markedly longer and the contents of H_2_O_2_, O_2_^−^, CAT and POD were also higher than those in WT lines, which suggested that the overexpression of *SaNramp6* enhanced the Cd uptake and accumulation in transgenic plants.

Taken together, we have functioned SaNramp6 in transgenic yeast and *A. thaliana* and the data presented in this study suggested that *SaNramp6* may be a critical Cd transporter responsible for Cd accumulation in *S. alfredii*. It was hard to place the functions of *SaNramp6* into specific categories such as uptake or translocation. The improved Cd uptake caused by *SaNramp6* may be due to the exertion of direct effects on several major pathways or may work in cooperation with other genes participating heavy metal uptake, transport, sequestration and detoxification. A similar case was reported recently, the uptake of Fe in roots by *NRAMP1* requires the partnership of another transporter, *IRT1*, in *A. thaliana* ^36^. Although the function of *SaNramp6* is still unclear, the gene appears to be related to the hyperaccumulator characteristic of *S. alfredii*. These findings will contribute to understanding the function of *Nramp* genes and provide experimental evidence and theoretical guidance for further studies.

## Methods

### Plant materials and growth conditions

A hyperaccumulating ecotype of *S. alfredii* was collected from the area of an old Pb/Zn mine in Quzhou City, Zhejiang Province, P. R. China. The plants were water-cultivated in an artificial climate chamber at 25 °C with a 16 h light/8 h dark cycle. The *S. alfredii* seedlings used for the stress treatment were asexual propagated to ensure consistency and grown in half-strength Hoagland-Arnon solution for about two weeks until relatively vigorous roots grew. For the expression analyses of target genes, plants were treated with 400 μM CdCl_2_ for 0 h, 0.5 h, 6 h, 12 h, 24 h, 48 h, 72 h, 96 h, 1 week and 2 week. Each treatment was replicated three times. All samples were quickly frozen in liquid nitrogen followed by storage at −80 °C until use.

*A. thaliana* (ecotype Columbia) was grown in a controlled environmental chamber at 22 °C under a long-day cycle (16 h light, 8 h dark), with a white light intensity of approximately 125 mmol·m^−2^·s^−1^ and 70% relative humidity. Overexpression lines, mutant and rescue lines, were selected for physiological assays. The seeds were surface sterilized and germinated on 1/2 Murashige and Skoog (MS) agar plates containing 25 mg·L^−1^ hygromycin. Whereafter, 30 d-old *Arabidopsis* seedlings were soaked in Hoagland-Arnon solution with or without 50 µM CdCl_2_ for 24 h and them used to measure the Cd^2+^ flux. For physiological assays, 30 d-old homozygous transgenic seedlings were soaked in Hoagland-Arnon solution with or without 30 µM CdCl_2_ for two weeks, and treatments for 0 h, 10 min, 20 min, 30 min, 1 h, 2 h, 4 h, 8 h, 12 h, 24 h, 48 h, 96 h, 1 week and 2 week used for Cd-uptake assay.

### RNA preparation, cDNA synthesis and DNA extraction

Leaves, stems and roots were harvested after each treatment, and all of the samples were frozen in liquid nitrogen and stored at −80 °C for analysis. Total RNA was isolated from the tissues using the Total RNA Purification Kit (NORGEN, Thorold, Canada). First-strand cDNA was then synthesized from 2 µg of total RNA by the Superscript RT III first-strand cDNA synthesis kit followed by RNase H (Invitrogen, Carlsbad, USA) treatment. Genomic DNA was isolated from seedling leaves using cetyltrimethyl ammonium bromide (CTAB) method as described by Murray and Thompson^37^.

### Cloning of *SaNramp6* gene

The full-length *SaNramp6* cDNA was amplified by reverse transcription-PCR (RT-PCR) and rapid amplification of cDNA ends-PCR (RACE-PCR).

The internal fragment of *SaNramp6* was isolated from *S. alfredii* using the specific primers *SaNramp6*-F and *SaNramp6*-R, which were designed according to transcriptome data^28^. To obtain the 3′-end cDNA and 5′-ready cDNA, four gene specific primers 3P1, 3P2, 5P1 and 5P2, were designed and synthesized based on the sequence of the cloned internal fragment. The cloning was performed as described by Wang *et al*.^38^. Additionally, the genomic sequence of *SaNramp6* was amplified by PCR using genomic DNA as the template with primers *SaNramp6*-F and *SaNramp6*-R. All of the primers are listed in Table 1.

**Table 1:** Degenerate and specific primers used in this work.

Primers	Sequence (5′-3′)	Description
*SaN1*-F	ATGGCATCAACTGTCGGAAACGC	qRT-PCR
*SaN1*-R	ACATGCCAATTCCACAGCGA	qRT-PCR
*SaNramp6*-F	ATGGCATCAACTGTCGGAAACGC	Gene specific amplification
*SaNramp6*-R	CTACTCTAAGACAGCTCTGCGTTGCGG	Gene specific amplification
*SaNramp6*-GF	CACCATGGCATCAACTGTCGGAAACGC	Gene specific amplification
*SaNramp6*-RT-F	TGTTTGGCGATTGTGCCAAG	qRT-PCR
*SaNramp6*-RT-R	ACATGCCAATTCCACAGCGA	qRT-PCR
*UBC9*-F	TGGCGTCGAAAAGGATTCTGA	qRT-PCR
*UBC9*-R	CCTTCGGTGGCTTGAATGGATA	qRT-PCR
*AtActin*-F	GCACCCTGTTCTTCTTACCG	qRT-PCR
*AtActin*-R	AACCCTCGTAGATTGGCACA	qRT-PCR
APL	AAGCAGTGGTATCAACGCAGAGTACGC(G)_10_	5′-RACE adapter primer
APS	AAGCAGTGGTATCAACGCAGAGT	5′-RACE universal primer
B 26	GACTCTAGACGACATCGA(T)_18_	3′-RACE adapter primer
B 25	GACTCTAGACGACATCGA	3′-RACE universal primer
5P1	ATGCAATTGAAACAAGAAAACCAGG	Reverse primer for 5′-RACE
5P2	ATGCCATCTATCGATCAAACTGTT	Reverse primer for 5′-RACE
3P1	AGGCTGGCGTGGTTGATACATGTG	Forward primer for 3′-RACE
3P2	TGTGCGAATCGGATCAAGTTT	Forward primer for 3′-RACE

### Bioinformatics analysis of *SaNramp6*

To compare the genomic structure, the genomic sequences of *A. thaliana Nramps* from GenBank were searched and the intron-exon structure was analyzed.

Translation and protein analyses of SaNramp6 were initially performed using ExPASy tools (http://www.expasy.org/tools/). CELLO v.2.5: subCELlular LOcalization predictor (http://cello.life.nctu.edu.tw/) and SOSUI version 1.11 (http://bp.nuap.nagoya-u.ac.jp/sosui/) were used to predict subcellular localization and transmembrane domains, respectively. For the multiple sequence alignment, Clustal Omega (http://www.ebi.ac.uk/Tools/msa/) was performed to align amino acid sequences first, and subsequently, the results were edited by GeneDoc. Additionally, a phylogenetic tree was constructed by MEGA 5.2 software using the Neighbour-joining method with 1,000 replicates based on amino acid sequences of the NRAMP proteins. The known NRAMP protein sequences from NCBI GenBank are shown in Table 2.

**Table 2:** Names and accession numbers of the Nramp protein family members.

Species	Name	Accession numbers
*Arabidopsis thaliana*	AtNramp1	NP_178198.1
*Arabidopsis thaliana*	AtNramp6	NP_173048.3
*Populus trichocarpa*	PtNramp1	XP_006368514.1
*Morus notabilis*	MnNramp6	EXB50420.1
*Triticum urartu*	TuNramp3	EMS65084.1
*Nicotiana tabacum*	NtNramp1	BAH66919.1
*Selaginella moellendorffii*	SmNramp	XP_002966634.1
*Solanum melongena*	SmNramp1	BAM34953.1
*Solanum torvum*	StNramp1	BAM34952.1
*Brassica juncea*	BjNramp	ACR16683.1
*Medicago truncatula*	MtNramp	XP_003602053.1
*Arachis hypogaea*	AhNramp1	AFQ37304.1
*Theobroma cacao*	TcNramp6	XP_007023419.1
*Oryza sativa* Japonica	OsNramp5	NP_001059312.1

### Expression pattern analysis

SYBR-based quantitative real-time PCR (qRT-PCR) reactions (SYBR premix EX Tag reagent, TaKaRa, Da Lian, China) were carried out in triplicate on a 7300 Real-Time PCR System (Applied Biosystems, CA, USA) according to the manufacturer’s instructions. Relative gene expression was estimated based on the 2^−ΔΔCt^ method, applying the geometric mean of two reference genes: *UBC9* and *TUB* ^39,40^. All of the primers for RT-qPCR are listed in Table 1.

### Expression vector construction

The open reading frame of *SaNramp6* was amplified by PCR using High Fidelity KOD-Plus DNA Polymerase (Toyobo, Japan) from the cDNA of *S. alfredii* using the specific primers *SaNramp6*-GF and *SaNramp6*-R (Table 1). The yeast expression vector pYES2.1 -SaNramp6 was generated using pYES2.1 TOPO® TA Expression Kit (Invitrogen, Carlsbad, USA). For subcellular location and plant expression vector, the purified PCR products were then cloned into the Gateway entry vector pENTR/D-Topo (Invitrogen, Carlsbad, USA) and positive clones were further sequenced to verify the direction and sequence accuracy. The sequence-verified plasmid was then recombined in pK7WGF2.0 and pH2GW7.0^41^ to generate pK7WGF2.0-*SaNramp6* and pH2GW7.0-*SaNramp6*, respectively.

### Subcellular localization of SaNramp6

The correct plasmid pK7WGF2.0-*SaNramp6* fused to the green fluorescent protein (GFP) was extracted by Plasmid Maxprep Kit (Vigorous, Beijing, China). Free vector p35S-GFP was used as control. *A. thaliana* protoplast isolation and transfection were performed as previously described^42^. The subcellular location of SaNramp6 was further investigated by transient expression in onion epidermal cells and *Nicotiana benthamiana* lower leaf epidermal cells as described by Liu *et al*.^43^ and Zheng *et al*.^44^, respectively. A LSM510 confocal laser scanning microscope (Carl Zeiss, Oberkochen, Germany) was used to observe the signals.

### Heterologous expression of *SaNramp6* in yeast

The *Saccharomyces cerevisiae* strain BY4742 *∆ycf1* (*MATα; his3Δ1; leu2Δ0; met15Δ0; ura3Δ0; YDR135c::kanMX4*) was a Cd-sensitive mutant, which lacked the ability to compartmentalize Cd into vacuoles^45^, was used to assess the Cd tolerance of *SaNramp6*. The yeast transformation was performed using the lithium acetate method^46^. Yeast ∆*ycf1* cells transformed with the empty pYES2.0 vector were used as controls. The transformed yeast cells were selected on synthetic defined medium lacking uracil. For complementation assays, a series of three 1:10 dilutions from each culture was spotted onto synthetic-galactose-uracil (SG-U) agar plates supplemented with 0, 15 and 20 µM CdCl_2_ and incubated at 30 °C for three days. The relative growth of transformants was determined by measuring the OD_600_ at 6 h intervals. For the Cd-uptake assay, yeast cells transformed with the empty or *SaNramp6* vector were grown for 48 h at 30 °C on SG-U supplemented with 5 µM CdCl_2_, then measured the Cd content.

### Detection of the *Arabidopsis Atnr* mutant by *Atnramp1*

To understand the functions of *SaNramp6*, we obtained mutant alleles from the SALK collection of sequence-indexed T-DNA insertions^47^. However, the mutant alleles of *AtNramp6* were not found. Therefore, a single insertion line (SALK_053236; *nramp1-1*) was confirmed for *SaNramp6* because *AtNramp6* and *AtNramp1* have similar genomic structure. A homozygous mutant was detected by PCR using the primers (LP/RP and universal primers BP) designed based on the T-DNA website (http://signal.salk.edu/tdnaprimers.2.html) (data not shown). The collected homozygous mutant seeds were air-dried and stored at 4 °C.

### Generation of transgenic *A. thaliana*

The recombinant plasmid pH2GW7.0-*SaNramp6* was introduced into *Agrobacterium tumefaciens* strain EHA105. *A. thaliana* ecotype Columbia plants were transfected by the floral dip method^48^. Positive transformants were selected based on hygromycin (Hyg, 20 μg·mL^−1^) resistance and confirmed by PCR and RT-PCR using the primers described above, *AtActin* (Table 1) was the internal control. Homozygous lines were identified by screening for non-segregation from each independent transformant (T_3_ generation).

### Physiological analysis of *SaNramp6* transgenic, mutant and rescue of mutant lines

Six overexpression lines (designated OE) with high transcriptional levels of *SaNramp6* and 26 rescue of *Arabidopsis* mutant lines were obtained in this study. Among them, the OE 2, OE 3, *Atnr*-N24 and *Atnr*-N28 lines were selected in the following study owing to their phenotypes.

To investigate the potential effects of *SaNramp6* in *A. thaliana*, *SaNramp6*-OE *A. thaliana* (OE 2 and OE 3), homozygous mutant *A. thaliana* (*Atnr*) and the rescue of the *Arabidopsis* mutants (*Atnr*-N24 and *Atnr*-N28), as well as wild type, were used for abiotic stress-related physiological analyses, including root length, peroxidase (POD) activity, catalase (CAT) content, H_2_O_2_ and superoxide anion accumulations, and measurements of the Cd^2+^ flux. All of the experiments were independently carried out three times.

As for analyzing peroxidase (POD) activity, catalase (CAT) content, H_2_O_2_ and superoxide anion accumulations, approximately 0.1 g of root tissue was ground in liquid nitrogen and placed it in 2-mL tubes. The extraction of these physiological indices used the appropriate kits according to the instruction manual (Comin, Suzhou, China).

The net Cd^2+^ fluxes in the roots of *Arabidopsis* were measured noninvasively by the Younger USA NMT Service Centre (Xuyue, Beijing) using the NMT system (NMT100 Series, Younger, USA LLC, Amherst, MA, USA). Prior to the flux measurement, the roots were equilibrated for 15 min in testing liquid (0.05 mM CdCl_2_, 0.1 mM KCl, 0.02 mM CaCl_2_, 0.02 mM MgCl_2_, 0.5 mM NaCl, 0.1 mM Na_2_SO_4_ and 0.3 mM MES, pH 5.7). Then, the transmembrane Cd^2+^ flux in roots was measured of different lines (120 µm to root apex) for 15 min by a Cd^2+^-selective microelectrode. All of the measurements were repeated at least six times independently.

### Cd concentration assay

To test the characteristic of *SaNramp6*′s Cd accumulation in *A. thaliana*, *SaNramp6*-OE *A. thaliana* (OE 3), homozygous mutant *A. thaliana* (*Atnr*), the rescue of the *Arabidopsis* mutant (*Atnr*-N28) and wild type were used in this experiment.

Roots and aerial parts were harvested individually for the Cd concentration analysis, and roots were resorbed by dipping in 1 mM EDTA for 30 min, and then washed three times with distilled water. All of the samples containing yeast cells for Cd determination were dried at 105 °C for 30 min, and then placed at 70 °C until they reached a constant weight. The dried samples were digested with a concentrated acid mixture of HNO_3_, HClO_4_, and H_2_SO_4_ (volume ratio = 4:1:0.5) at 250 °C for 8 h. The metal concentration in the digested solution was determined by atomic absorption spectrometry (M6; SOLLAR) and an inductively coupled plasma-mass spectrometer (ICP-MS; NexION 300; PerkinElmer) after dilution.

### Data processing

Data were exhibited as the means ± standard deviations (SDs) of at least three independent biological experiments. Statistical analysis was performed using SPSS 17.0 statistics software. To test significant changes in mRNA relative expression and Cd concentration, time and Cd treatment were regarded as the main factors. Tukey- HSD method was used to correct all P-values of these multiple comparisons. In addition, one asterisk (*) or two asterisk (**), significantly different from control at P = 0.05, 0.01, respectively.

The translocation factor for Cd within a plant was expressed by the concentration in the aerial parts (µg·g^−1^DW)/the concentration in the roots (µg·g^−1^DW), which showed the Cd-translocation properties from roots to aerial parts^49^.

## Electronic supplementary material

Supplementary Information
