# Supplementary material for: Sedum alfredii SaNramp6 Metal Transporter Contributes to Cadmium Accumulation in Transgenic Arabidopsis thaliana
Source: Sci Rep. 2017 Oct 17;7:13318. doi: 10.1038/s41598-017-13463-4 (PMC5645334; doi:10.1038/s41598-017-13463-4)
Supplement: Supplementary file 1 — Supplementary Information [file 41598_2017_13463_MOESM1_ESM.pdf]

Sedum alfredii SaNramp6 Metal Transporter Contributes to Cadmium Accumulation in Transgenic *Arabidopsis thaliana*

Shuangshuang Chen [a,b,1], Xiaojiao Han[a,b,1], Jie Fang[a,b,c,1], Zhuchou Lu[d], Wenmin Qiu [a,b], Mingying Liu[a,b], Jian Sang[a,b], Jing Jiang[a,b], and Renying Zhuo[a,b*]

a, State Key Laboratory of Forest Genetics and Breeding, Xiangshan Road, Beijing, 100091, P.R. China

b, Key Lab of Tree Genomics, The Research Institute of Subtropical of Forestry, Chinese Academy of Forestry

c, Chemical Biology Center, Lishui Institute of Agricultural Sciences, Lishui, Zhejiang Province, 323000, China

d, Biotechnology Research Center of China Three Gorges University, Yichang, Hubei 443002, China

[1] These authors contributed equally to this work.

* Corresponding authors

E-mail address: zhuory@gmail.com (R.-y. Zhuo)

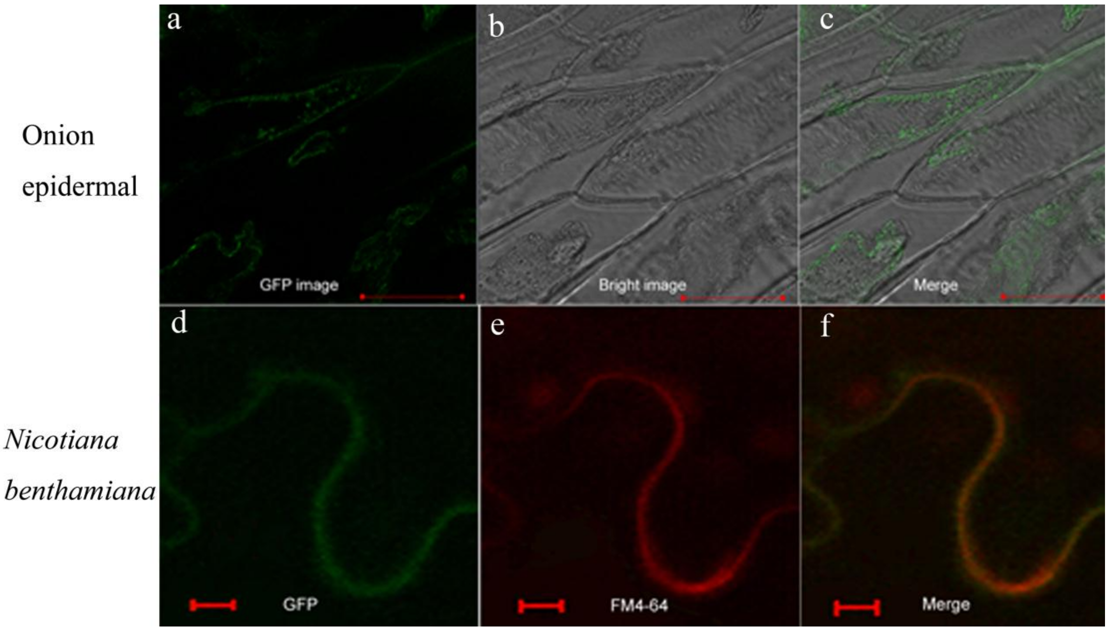

Supplementary Figure S1 Subcellular localization of SaNramp6 in onion epidermal cells and *Nicotiana benthamiana* leaf epidermal cells. (a-c) Subcellular location of SaNramp6-GFP in onion epidermal cells, Scale bar=50 μm. (d-f) Confocal images of a tobacco leaf epidermal cell showing colocalization of SaNramp6-GFP (d), FM4-64 (five minutes after staining, e) and the merging of d and e (f), Scale bar=10 μm.

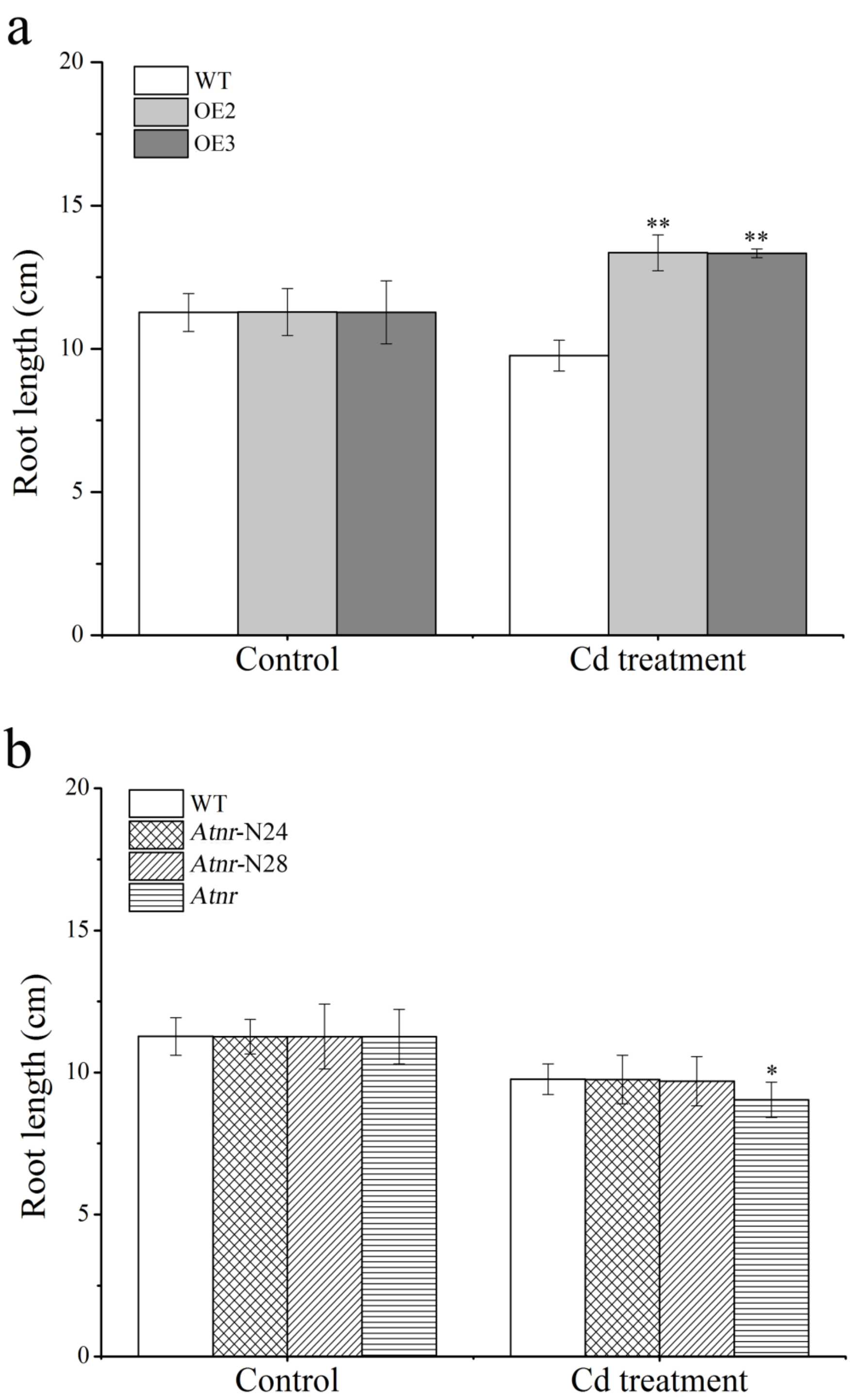

Supplementary Figure S2 Root length of different four lines after treatment with or without 30 μM Cd - WT (wild type); OE 2 and OE 3 (overexpression lines); *Atnr* (mutant line); *Atnr*-N24 and *Atnr*-N28 (rescue lines). Bars indicate means ± standard deviations (SDs) of at least three independent biological experiments. One or two asterisks indicate a significant difference at $P < 0.05$ or $P < 0.01$ from wild type.
